# Exosomes derived from mir-337-3p over-expressing tendon stem cells protect against apoptosis of tenocytes via targeting caspase3

**DOI:** 10.1186/s12891-024-07691-9

**Published:** 2024-07-19

**Authors:** Qing An, Zipeng Zhou, Chang Xu, Qiang Xiao

**Affiliations:** Department of Hand Surgery, The First Affiliated Hospital of JinZhou Medical University, No.2, Renmin Street, Section 5, Guta District, Jinzhou City, Liaoning Province 121000 China

**Keywords:** Tendon, Tendon stem cells, Tenocyte, Exosomes, miR-337-3p, CASP3

## Abstract

**Background:**

Tendons are important dense fibrous structures connecting muscle to bone, and tendon stem cells (TDSCs) affect their repair and regeneration. The role of TDSC-derived exosomes (TDSC-Exos) is still being unexplored; therefore, this study aimed to investigate the protective effect of TDSC-Exos on tenocytes.

**Methods:**

The TDSCs and tenocytes were all derived from *Sprague Dawley* (SD) rats. The expression of positive and negative markers of TDSCs were detected by flow cytometry, and the multi-differentiation ability was also detected to identify TDSCs. Exos were derived from TDSCs using ultracentrifugation; furthermore, Exos enriched with microRNA(miR)-377-3p were generated from TDSCs stably overexpressing miR-377-3p after transfection, identified with transmission electron microscopy (TEM), western blot and PKH26 staining assay. Moreover, the cell functions of tenocytes were evaluated by MTT, EdU, transwell, and flow cytometry. Dual luciferase reporter and RNA pull-down assays were used to verify the binding sites of miR-337-3p and caspase3 (CASP3) predicted by Targetscan.

**Results:**

Exos (miR-337-3p) were taken up by tenocytes, and promoted the proliferation, migration, and invasion and suppressed the apoptosis of tenocytes in a dose-dependent manner. Bioinformatics analysis showed that CASP3 was a target of miR-377-3p, which was further verified by luciferase and RNA pull-down assays. Moreover, over-expressed CASP3 reversed the effects of Exos (miR-337-3p) on cell functions of tenocytes.

**Conclusions:**

Our findings suggest that Exos derived from miR-337-3p over-expressing TDSCs could potentially protect against tenocyte apoptosis by regulating CASP3. This novel therapeutic approach holds promise for the treatment of tendon injury, offering a glimmer of hope for improved patient outcomes.

**Supplementary Information:**

The online version contains supplementary material available at 10.1186/s12891-024-07691-9.

## Introduction

Tendons are a type of connective tissue that possesses intricate mechanical functions and can transform muscle contraction into joint movement and transmit power from muscle to bone, which is essential for maintaining body balance [1]. Recent reports suggested an increasing trend in the incidence of tendinopathy, making tendon-related health problems prevalent [2]. The available treatment modalities are at varying risk of complications, in addition to the unresolved problem of re-rupture [3, 4]. These shortfalls make it imperative to unveil the mechanism of tendon protection further.

Tendon stem cells (TDSCs) have been shown to exhibit characteristics of stem cells such as cell cloning colonies, self-replication, proliferation, and polymorphic differentiation both in vivo and in vitro [5, 6]. Moreover, studies have also verified that these TDSCs were able to form “tendon-like tissues” in nude mice [7, 8], cementing the fact that TDSCs are beneficial in tendon repair and reconstruction. Exosomes (Exos) are nanosized vesicles enclosed by lipid bilayer membranes secreted by various cells and range in size between 30 nm and 100 nm [9]. They have been reported to carry a variety of substances, including mRNAs, miRNAs, proteins, and lipids, and play an important role in cell-to-cell communication [9]. Recent findings indicated that Exos plays a role in the pathological and physiological processes of certain diseases, such as tendon regeneration [5]. Similarly, bone marrow mesenchymal stem cell-derived exosomes (BMSC-Exos) were found to enhance rotator cuff tendon-bone healing in rats by stimulating angiogenesis and reducing inflammation [10], while human umbilical cord mesenchymal stem cell-derived exosomes (HUMSC-Exos) were found to promote tendon regeneration via PTEN/mTOR/TGF-β1 signaling pathway [11]. However, the role of tendon-derived stem cell-derived exosomes (TDSC-Exos) is rarely discussed.

Exosomal miRNAs have proven to play an important role in the process of tissue repair and regeneration, such as promoting angiogenesis, mediating inflammation and regulating cellular function [12]. The miRNAs are endogenous non-coding small molecules with a length of about 20 nt, are widely distributed in organisms’ genomes [13], and have been found to play important roles in regulating tenocytes [14]. Song et al. suggested that TDSC-Exos enhanced tenon repair through miR-144-3p-regulated tenocyte proliferation and migration [12]. Therefore, it is of great significance to explore the expression of miRNA to better explain the therapeutic effect of TDSC-Exos on tendon injury. The miR-337-3p is a regulatory factor closely related to human tumors and has been found to inhibit the proliferation of several human tumor cells by regulating downstream target molecules [15, 16]. Furthermore, elevated miR-337-3p is also reported to facilitate tendon healing by targeting downstream genes to regulate the balance between chondro-osteogenic and tenogenic differentiation of tendon progenitors in rats [17]. Whether miR-337-3p participates in the regulation of protecting tenocytes against apoptosis remains unclear.

In this study, we speculated that miR-337-3p in TDSC-Exos may protect against tendinopathy in vitro. To test our hypothesis, we extracted Exos from TDSCs with overexpressed miR-337-3p, and these Exos were taken up by tenocytes. The protecting effects of Exos over-expressing miR-337-3p on tenocytes were evaluated by detecting the cell proliferation, migration, invasion, and apoptosis. This study may offer insights into the future use of Exos in treating tendon injuries.

## Materials and methods

### Experimental animals

Six eight-week-old male SD rats with body weight of 250–300 g were provided by Jiangsu Academy of Agricultural Sciences (License Number: SCXK(Su)2018-0024). The treatment of animals in the experiment is in accordance with the guide for the Care and Use of Laboratory Animals. The animal use protocol has been reviewed and approved by the Animal Ethical and Welfare Committee(AEWC)[Approval No. MDKN-2021-063].

### Isolation of TDSCs

The rats were euthanized by injection of pentobarbital sodium (P276000, 100 mg/kg, i.p. AmyJet Scientific, China). Then the middle part of the patellar tendons were excised from healthy rats and washed in sterile phosphate-buffered saline solution (PBS) with 10% PSN (500 µg/mL penicillin, 500 µg/mL streptomycin, and 1000 µg/mL neomycin) for 5 min. After removing the aponeurosis from the surface of the tendon, the tendon was cut into small pieces (1cm^3^) and placed in a petri dish [7]. 0.3% type I collagenase (17,100,017, Gibco, Grand Island, NY, USA) was added for digestion, followed by filtered by 70 μm cell filter; the single-cell suspension obtained were centrifugated and resuspended on basic medium (100 U/mL penicillin, 100 mg/L streptomycin). The cell suspension was then inoculated with 50 cells/cm^2^. After 3 days of culture, the cells were washed with sterile PBS to remove the unadhered cells. After 7–10 days of culture, the growth cells were digested with 0.25% trypsin (25,200,072, Gibco, Grand Island, NY, USA) and labeled as primary cells. The cells in the third generation were used for stem cell identification and subsequent experiments.

### Identification of TDSCs

Identification of TDSCs was performed as previously described [8, 18]. The expression of stem cell surface makers in TDSCs was analyzed by flow cytometry. TDSCs at passages three were incubated with fluorescein isothiocyanate-conjugated antibodies (anti-CD90: ab225, 1/200, 0.1 µg for 10^6^ cells; anti-CD105: ab221675, 1/500; anti-CD44: ab189524, 1/40; anti-CD106: ab223982, 1/500; and anti-CD11B: ab184308, 1/70.) (Abcam, Cambridge, MA, USA) through CytoFLEX S flow cytometry (Beckman Coulter, Miami, FL, USA). Cell morphology was observed by inverted phase contrast microscope (Eclipse Ts2, Nikon, Tokyo, Japan).

### Cell transfection of TDSCs

The miR-337-3p overexpression vector (miR-337-3p mimic) and its negative empty vector (nc mimic), shRNA targeting miR-337-3p (miR-337-3p inhibitor) and nc inhibitor were designed and synthesized by GenePharma (Shanghai, China), and the synthesized precursor sequence of miR-337-3p was inserted into the lentiviral vector. Lentiviral packaging was performed in HEK293T cells. After 48 h, the virus supernatant was collected for filtration and concentration. TDSCs were cultured in 6-well plates at a concentration of 2 × 10^5^ cells per well. The concentrated lentiviral supernatant was mixed with 8 µg/mL Polybrene and then added to the TDLSCs culture medium.

### Adipogenic differentiation of TDSCs

The TDSCs cultured to the third generation were inoculated into 6-well plates at a concentration of 4 × 10^3^cells/cm^2^. After cell growth and fusion, the basal medium was changed into lipid induction medium containing 500 nmol/L dexamethasone, 500 µmol/L isobutylmethyl xanthine, 50 µmol/L indomethacin, and 10 mg/L insulin (Sigma, St. Louis, MO, USA). On the 21th day, the cells were stained with oil red O to detect the formation of lipid droplets, and the morphological changes of cells were observed under inverted phase contrast microscope (Eclipse Ts2, Nikon, Tokyo, Japan).

### Osteogenic differentiation of TDSCs

The TDSCs in the third generation reached 80% confluence in 6-well plates were incubated with osteogenic medium (100 nM dexamethasone, 50 mg/mL ascorbic acid, and 5 mmol/L b-glycerophosphate; St. Louis, MO, USA) for 14 days, and the medium was changed every 3 days. Alizarin red S (ARS) staining (C0138, Beyotime, Shanghai, China) was performed after 14 days of incubation with osteogenic medium. TDSCs were fixed with 10% formaldehyde buffer for 10 min, then rinsed with distilled water for 3 times, 0.1% alizarin red-Tris-HCl dye solution (pH8.3) was added, and stained at 37 ℃ for 30 min. After rinsed with distilled water, the positive cells were observed under inverted phase contrast microscope (Eclipse Ts2, Nikon, Tokyo, Japan).

### Isolation and identification of TDSC-Exos

After reaching 70% confluency in 150 cm^2^ cell culture dishes, TDSCs were rinsed three times with PBS and then cultured in low glucose DMEM with 10% FBS (Exos free) for 48 h. The conditioned medium was collected for the Exo isolation. Ultracentrifugation was used to separate and extract Exos [19]. To remove cell debris, the obtained medium was centrifuged at 300×g for 10 min, 2000×g for 10 min and 10,000 g for 60 min at 4 °C. After centrifugation, the supernatant was filtered with a 0.22 μm filter (Millipore, Billerica, MA, USA) to remove the microparticles. Subsequently, the supernatant was ultracentrifuged at 100,000×g for 70 min. Exos at the bottom of the centrifuge tube were resuspended in PBS, and then were centrifuged at 100,000×g for 1 h at 4 °C. In order to obtain purified Exos, we removed the supernatant and added 200 µL of PBS to resuspend the pellet (1 mL of PBS containing 0.8 mg of Exos).

In order to identify isolated Exos, transmission electron microscope (TEM; JEOL, Tokyo, Japan) was used to observe the morphology of Exos. Nanoparticle tracking analysis (NTA) is used to determine the size distribution and particle concentration of Exos. The Exos were loaded onto copper grids coated with Formvar (Structure Probe). The grids were contrasted using 2% uranyl acetate, dried and then captured using a digital camera (Olympus, Tokyo, Japan). Specific markers of Exos (CD9, CD81, and HSP70) were detected by western blot method. Three concentrations of Exos (miR-337-3p) were prepared for follow-up studies: Exos (miR-337-3p) were diluented with PBS at final concentrations of 25, 50–100 µg/mL, respectively.

### Isolation of tenocytes

After SD rats were sacrificed, the mid-substance tissue of the rat patellar tendon was transferred to a 100 mm culture dish containing PBS, and epitenon membrane sheet was then removed. The tendon tissue was cut into small pieces and placed in 6-well culture plates. DMEM containing 10% FBS and 1% penicillin/streptomycin was added. The medium was changed every three days. After day 7–10, cells began to emerge from the pieces. The adherent cells were trypsinized and cultured as passage zero. Cells in passage five were used in the following experiments.

### Cell transfection of tenocytes

The CASP3 overexpression vector (oe-CASP3) and its negative empty vector (oe-nc) were all bought from GenePharma (Shanghai, China). The transfection process was the same as that of TDSCs.

### Exos uptake assay

In order to observe the Exos intake of tenocytes, lipophile fluorescent dye PKH26 (PKH26GL, Sigma, St. Louis, MO, USA) was used to stain Exos, then the free dye was removed by dialysis and added to a 6-well plate. After incubation for 4 h, the cells were washed with PBS for 3 times and fixed in polyformaldehyde for 15 min and stained with DAPI for 5 min. The slide was mounted with ProLong Gold Antifade Reagents, and the internalization of the Exos was observed by fluorescence microscope (Olympus, Tokyo, Japan).

### Western blot

After cell lysis, protein concentration was detected by BCA method (P0009, Beyotime, Shanghai, China) and adjusted to the same level. The lysis products were separated by 10% polyacrylamide gel electrophoresis and transferred to PVDF membrane (Millipore, Billerica, MA, USA) with gel imprinting. The membranes were sealed in 5% skim milk powder and crossbred with primary antibodies of CD9 (ab236630, 1/1000, Abcam, Cambridge, MA, USA), CD81 (ab79559, 2 µg/mL, Abcam, Cambridge, MA, USA), HSP70 (ab2787, 1/1000, Abcam, Cambridge, MA, USA), cleaved-caspase-3 (AB3623, 1/1000, Sigma, St. Louis, MO, USA), Bax (ab32503, 1/1000, Abcam, Cambridge, MA, USA), and Bcl2 (ab194583, 1/500, Abcam, Cambridge, MA, USA), respectively, and incubated overnight at 4 ℃. The strips were incubated in the secondary antibody at room temperature for 2 h, and ECL was developed for color. The gray values of the strips were analyzed using GAPDH as internal reference.

### qRT-PCR

qRT-PCR was conducted to measure the expression levels of miR-337-3p and CASP3. Total RNA of each Exos (or culture medium) was extracted using an miRNeasy Mini Kit (QIAGEN, Duesseldorf, Germany) and cDNA was synthesized using reverse transcription kit (Takara). qRT-PC was conducted using a 7500 Real‐Time PCR Detection System (Applied Biosystems) with a Power SYBR Green Master Mix (Roche, Basel, Switzerland) according to the following settings: 95 °C for 10 min, 40 cycles of 95 °C for 15 s and 60 °C for 1 min. Relative gene expression was calculated using the 2^−ΔΔCt^ method. The miR-337-3p primers were 5′-AGTGTAGTGAGAAGTTGGGGGG-3′ (forward) and 5′-TTGAAGGGGGTGAAGAAAGGCA-3 (reverse). The CASP3 primers were 5′-CTCGCTCTGGTACGGATGTG-3′ (forward) and 5′-TCCCATAAATGACCCCTTCATCA-3′ (reverse).

### MTT

After incubation for 12, 24 and 48 h, 20 µL MTT solution (5 g/L, C0009, Beyotime, Shanghai, China) was added to each well, and the culture was continued for 4 h. After the supernatant was absorbed, 150 µL dimethyl sulphoxide was added, and the absorbance (A) at 490 nm of each well was measured by after shaking at low speed for 10 min. The A value represented the proliferation activity of the cells.

### EdU assay

The whole experiment was carried out according to the instructions of BeyoClick^TM^EDU-594 cell proliferation detection Kit (C0078, Beyotime, Shanghai, China). Five fields were randomly selected under the microscope (Olympus, Tokyo, Japan) to take photos and count, and the results were calculated. The test was repeated three times.

### Migration and invasion analysis of tenocytes

The transwell co-culture system with pore size of 8.0 μm (Corning Inc., NY, USA) was established in six-well plates. Number of migrated cells: 200 µL cell suspension was added to the upper chamber of transwell at the concentration of 4 × 10^4^ cells, and then 500 µL culture medium containing 10% FBS was added to the lower chamber for culturing for 24 h. After that, cells in lower chamber were fixed with methanol for 20 min, and washed with PBS 3 times. After 35 min of Giemsa staining, 5 fields were randomly selected under the microscope (Olympus, Japan) to take photos and count, and the average value was the final result, resulting in a total magnification of 200x, to document the migratory response. Number of invaded cells: 80 µL matrix gel was coated in a small chamber and incubated in 37 ℃ incubators for 30 min before cells were inoculated. The other steps are the same as the detection of cell migration.

### Dual luciferase reporter assay

The 3’-UTR of CASP3 containing the predicted miR-337-3p binding sites was synthesized and then cloned into a modified ver sion of pcDNA3.1(+) containing a firefly luciferase reporter gene at a position downstream of the luciferase reporter gene to construct an CASP3 wild type (wt) luciferase reporter plasmid. A Site Directed Mutagenesis Kit (SBS Genetech Co., Ltd) was then used to mutate the miR-337-3p binding site in the 3’-UTR of CASP3 and named as CASP3 mutant type (mut) luciferase reporter plasmid. All constructs were confirmed by DNA sequencing. Tenocytes were inoculated in 24-well plates for 24 h and starved for 30 min. After that, miR-337-3p over-expression plasmid, control plasmid and CASP3 3’-UTR wt and mut plasmids were co-transfected into tenocytes. 48 h later, PLB lysate was lysed for 15 min to extract suspension. Luciferase activity was measured, relative fluorescence intensity was analyzed, and the calculation results were obtained.

### RNA pull-down assay

Streptavidin beads were pretreated with 1% RNase-free BSA and 0.5 g/L yeast tRNA. The biotin probe and control probe of miR-337-3p were mixed and incubated with magnetic beads, respectively. After the cells were cleaned by PBS, the cells were lysed in ice bath, and then centrifuged to take 50 µL of the supernatant as input. The remaining supernatant was divided into two tubes, and then the lysates were combined with the prop-magnetic bead complex. Then, the complex of magnetic beads and RNA was extracted with Trizol, and the expression level of CASP3 was detected by qRT-PCR.

### Statistical analysis

The experimental data were analyzed by GraphPad Prism software version 8.3 (GraphPad, USA), and the data operation between the two groups was represented by mean ± SD and compared with t test. One-way ANOVA was used to compare the mean of multiple groups. Tukey’s post hoc test was used to compare pairwise comparisons between groups. *P*<0.05 means the difference is statistically significant.

## Results

### Characterization of TDSCs

**Fig. 1:**
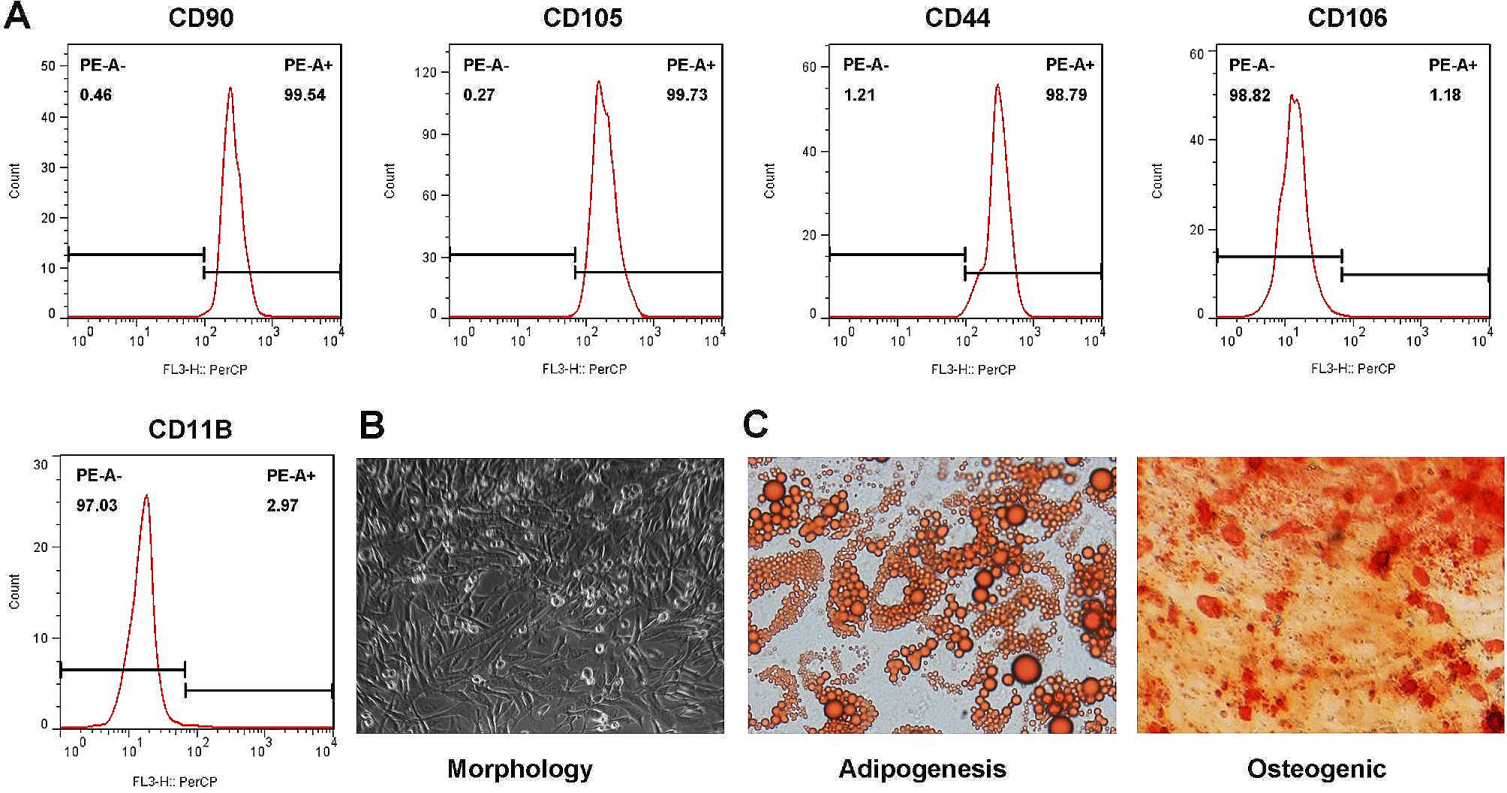
Characterization of TDSCs. (**A**) Surface markers including CD90, CD105, CD44, CD106 and CD11B measured by flow cytometry assay. (**B**) Morphology of TDSCs. (**C**) The potential of multilineage differentiation of TDSCs

Figure 1A shows the expression levels of positive markers CD90 (99.54%), CD105 (99.73%), and CD44 (98.79%), as well as negative markers CD106 (1.18%) and CD11B (2.97%). Additionally, TDSCs exhibited a typical fusiform morphology (Fig. 1B), and with an ability to differentiate into adipocytes and osteoblasts (Fig. 1C).

**Fig. 2:**
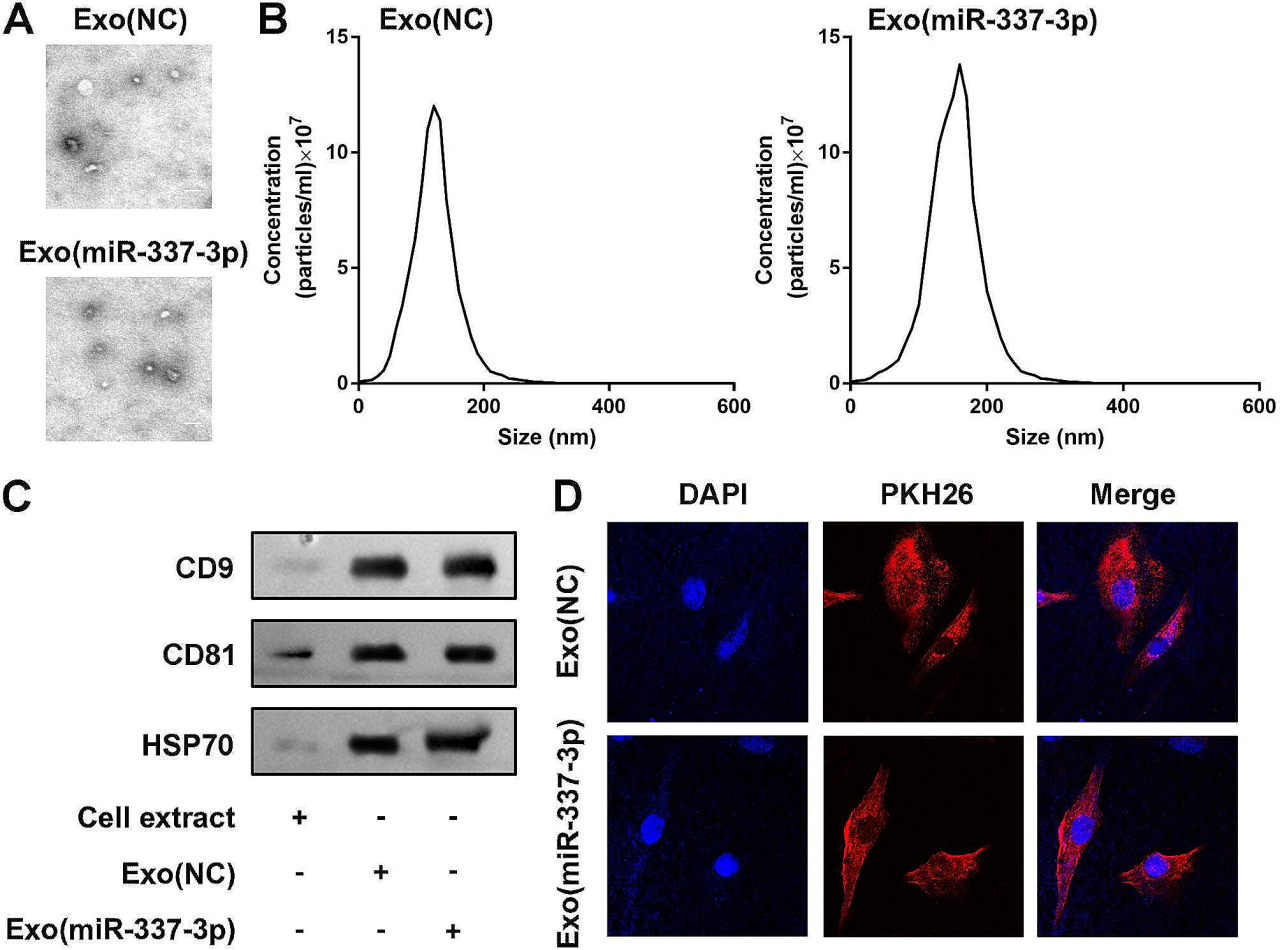
Characterization of TDSC-Exos. (**A**) Morphology, and (**B**) particle size distribution observed under TEM. (**C**) Western blot analyses of Exos-specific protein expression. (**D**) Images of PKH26-labeled TDSC-Exos in tenocytes

### Characterization of TDSC-Exos

Exos derived from TDSCs pre- and post-transfection displayed round vesicular structures (Fig. 2A), where the average size of Exos in the negative control group was 110 nm, while 150 nm in the miR-337-3p over-expressing group (Fig. 2B). Exos-specific proteins such as CD9, CD81, and HSP70 showed increased expression in TDSC-Exos compared to TDSCs, with the highest levels observed in miR-337-3p over-expressed TDSC-Exos (Fig. 2C). TDSC-Exos, labeled with lipophilic fluorescent dye PKH26, were observed surrounding the cell nucleus, suggesting the uptake of TDSC-Exos by tenocytes (Fig. 2D).

### Mir-337-3p over-expressing TDSC-Exos (Exos (miR-337-3p)) exerted protecting effects on tenocytes

**Fig. 3:**
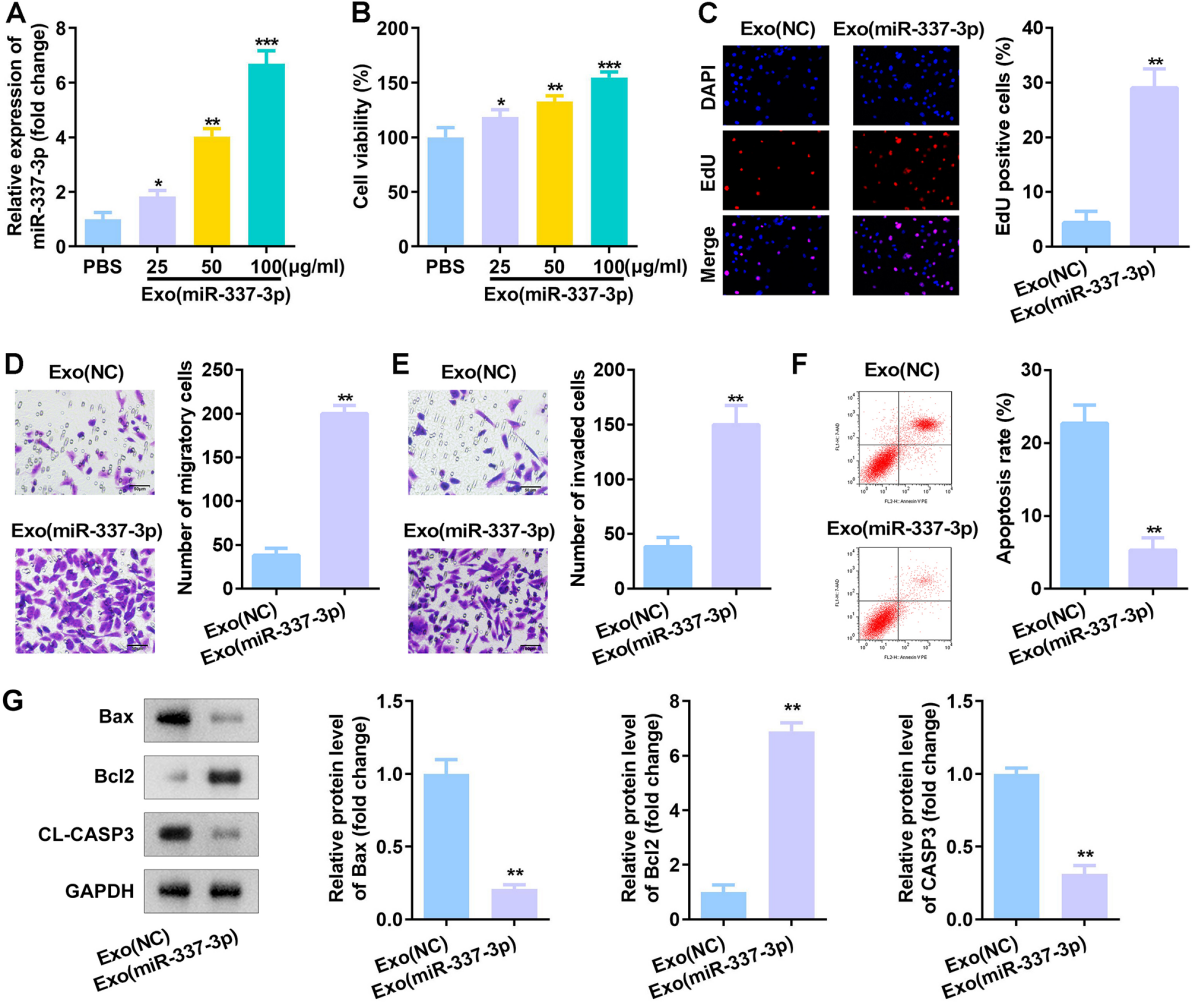
miR-337-3p over-expressing TDSC-Exos (Exos (miR-337-3p)) exerted protecting effects on tenocytes. (**A**) RT-qPCR analyses of miR-337-3p expression in Exos (miR-337-3p) at different concentrations. (**B-C**) Cell proliferation of tenocytes treated with Exos (miR-337-3p) measured by MTT and EdU assays. (**D-E**) Transwell assay was conducted to evaluate migration and invasion of tenocytes treated with Exos (miR-337-3p). (**F-G**) Cell apoptosis was analyzed from flow cytometry and western blot methods. ^*^p < 0.05, ^**^p < 0.01, ^***^p < 0.001

Results showed an elevated miR-337-3p expression with an increase in Exos (miR-337-3p) concentration (Fig. 3A). Moreover, Exos (miR-337-3p) also promoted cell viability of tenocytes in a dose-dependent manner (Fig. 3B). Exos (miR-337-3p) at 100 µg/mL were used for subsequent experiments, and Exos (miR-337-3p) promoted cell proliferation (Fig. 3C), migration (Fig. 3D), and invasion (Fig. 3E) of tenocytes. Moreover, tenocytes apoptosis was also suppressed by Exos (miR-337-3p) accrording to the results of flow cytometry and western blot methods (Fig. 3F-G).

**Fig. 4:**
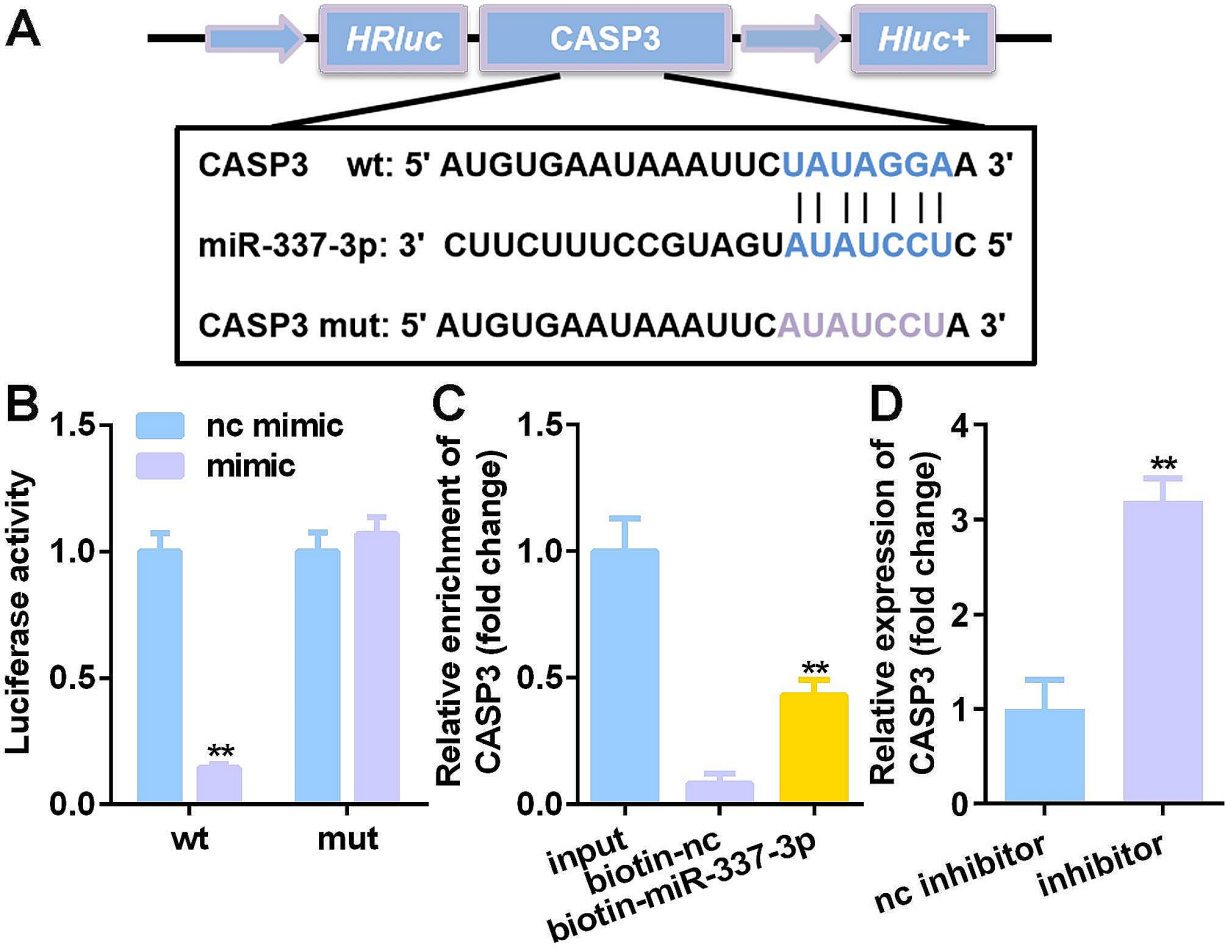
CASP3 was a downstream gene of miR-337-3p. (**A**) Bioinformatics predicted the binding sites between miR-337-3p and CASP3. (**B**–**C**) The association between CASP3 and miR-337-3p was confirmed by a dual-luciferase reporter as well as RNA pull-down assays. (**D**) RT-qPCR analysis for the expression of CASP. ^**^p < 0.01

### CASP3 was a downstream gene of miR-337-3p

A potential binding site between miR-337-3p and CASP3 has been reported in an online tool Targetscan (Fig. 4A) [20]. The luciferase activity of tenocytes co-transfected with wild-type 3’URT CASP3 and miR-337-3p mimic was evidently decreased compared to in mutant group (Fig. 4B). Moreover, the RNA pull-down assay results revealed the enrichment of CASP3 on biotin-miR-337-3p probe (Fig. 4C), whereas the CASP3 was found significantly up-regulated following knocking down the miR-337-3p expressions (Fig. 4D).

### CASP3 alleviated effects of Exos (miR-337-3p) on celluar functions of tenocytes

**Fig. 5:**
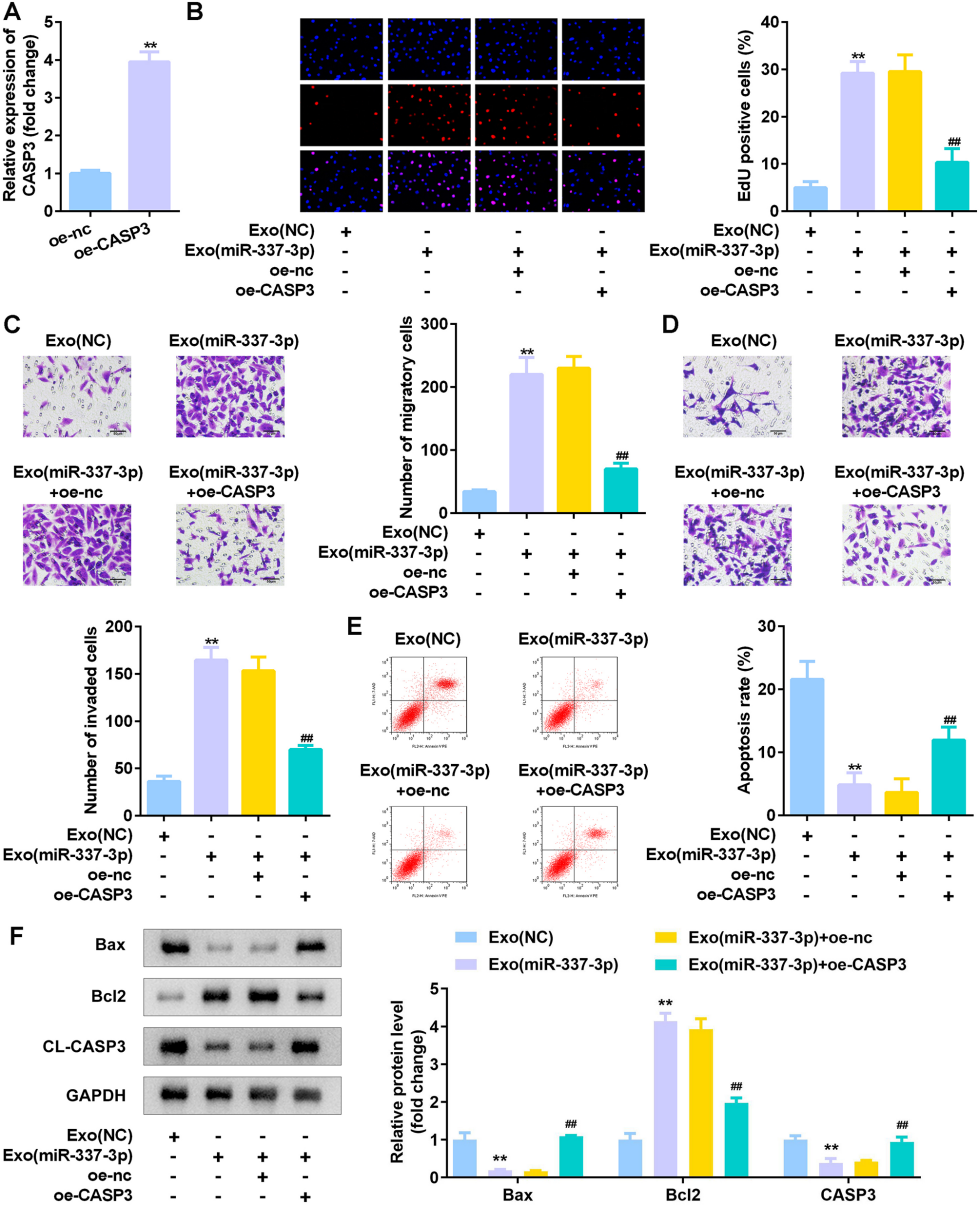
CASP3 alleviated effects of Exos (miR-337-3p) on cell functions of tenocytes. (**A**) RT-qPCR analyses of CASP3 expression in tenocytes transfected with oe-CASP3. (**B**) Cell proliferation of tenocytes treated with Exos (miR-337-3p) after transfection was measured by MTT and EdU assays. (**C**–**D**) A Transwell assay was conducted to evaluate the migration and invasion of tenocytes treated with Exos (miR-337-3p) after transfection. (**E-F**) Cell apoptosis was analyzed from flow cytometry and western blot methods. ^**^p < 0.01, compared with oe-nc and Exo (nc) groups. ^##^*p* < 0.01, compared with Exo (miR-337-3p) + oe-nc group

Then CASP3 levels were succefully up-regulated in tenocytes according to the qPCR analysis (Fig. 5A). Moreover, Exos (miR-337-3p) promoted proliferation (Fig. 5B), migration (Fig. 5C), and invasion (Fig. 5D), and suppressed tenocytes apoptosis (Fig. 5E-F). The accelerated proliferation, migration, invasion, and apoptosis inhibition induced by Exos (miR-337-3p) were reversed by up-regulation of CASP3 in tenocytes treated with Exos (miR-337-3p) (Fig. 5B–F).

**Fig. 6:**
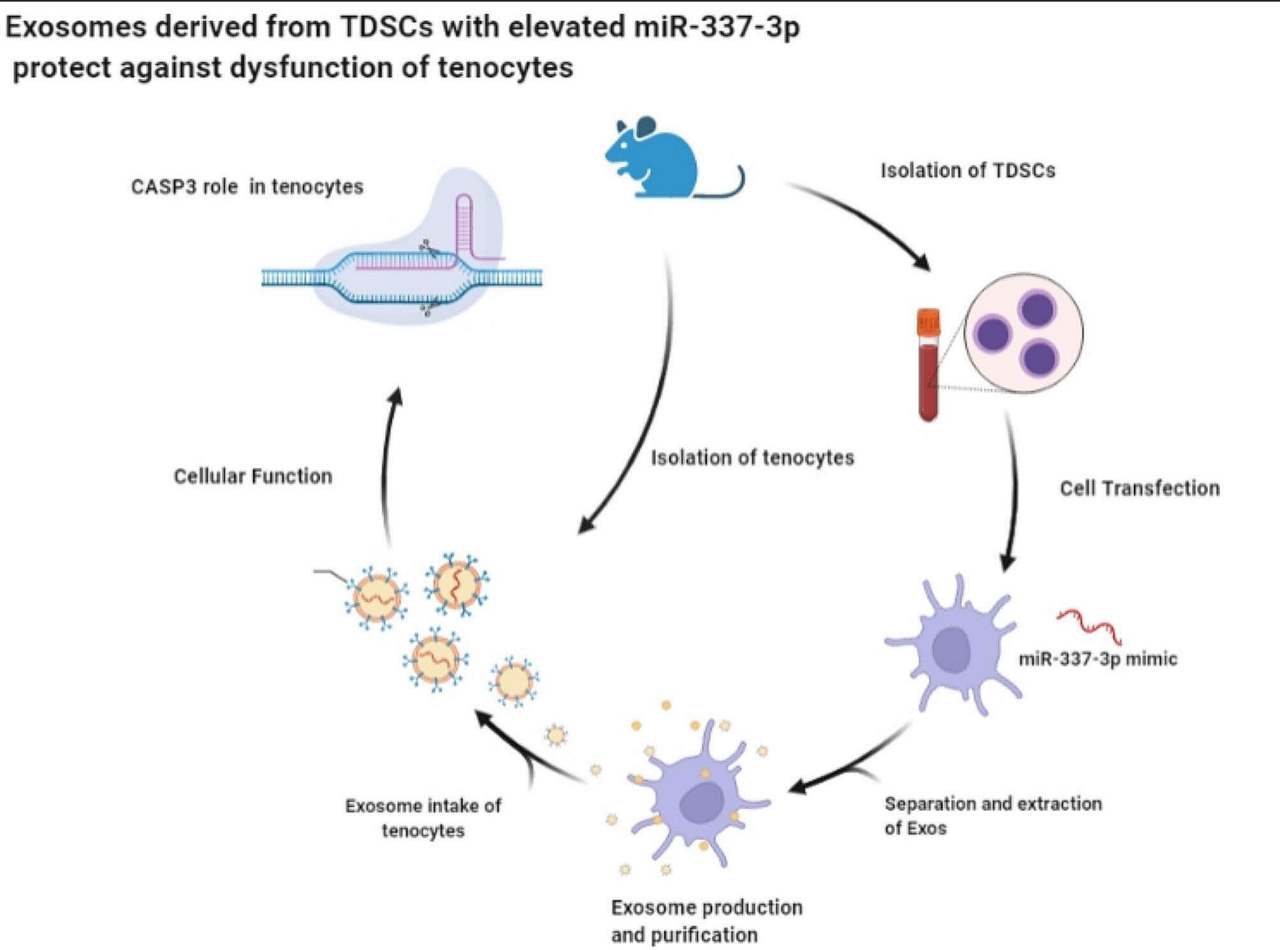
Graphical abstract

## Discussion

Tendons connect muscles to bones, and their normal structure is rarely restored following injury due to the formation of fibrous scarring that destroys tissue structure. Tendon injury treatment is currently hindered and limited in progress due to a lack of understanding of the pathogenesis and healing mechanism of tendon injury. In this study, Exos (miR-337-3p) were taken up by tenocytes, and promoted the proliferation, migration, and invasion and suppressed the apoptosis of tenocytes. This is related to the regulation of CASP3 modulated by Exos (miR-337-3p) (Fig. 6 graphical abstract).

Cell-based therapies recently have provided a novel alternative strategy for tendon repair and reconstruction, including TDSCs, bone marrow mesenchymal stem cells (BMMSCs) and adipose mesenchymal stem cells (AMSCs) [17, 21, 22]. Research indicates that TDSCs exhibit significant chondrogenic and osteogenic differentiation potential when compared to other types of stem cells. Moreover, TDSCs have been found to reshape tendons following transplanting them into tendon defects as exogenous or endogenous seed cells for tissue engineering tendon regeneration [23], where tendinous differentiation of TDSCs was induced to play the role of remodeling injured tendons. Similarly, in tendon injury repair, a small number of TDSCs can be activated by adding corresponding cytokines to promote their proliferation and differentiation to reduce scar healing and promote tendon repair [24, 25]. Therefore, understanding the mechanism of TDSCs to restore tendons might be a novel perspective in tendon remodeling.

Exos are extracellular vesicles containing bioactive molecules like lipids, proteins, and nucleic acids, and can be released by any cell type and widely distributed in all biological fluids, including blood, saliva, sweat, and urine [9]. They can participate in various physiological processes such as intercellular communication, mammalian reproduction, and immune response, and also play an important role in metabolism and the pathological progression of diseases like cardiovascular, neurodegeneration, and cancer, making them appealing as natural non-invasive biomarkers; thus, considered as a potential tool for clinical diagnosis and treatment. Recent evidence indicated that Exos improved tendon healing through various mechanisms; e.g., BMMSC-derived Exos were found to contribute to tendon regeneration by promoting proliferation and migration of tendon stem/progenitor cells [5]. Similarly, BMMSC and TDSC-derived Exos were also found to facilitate tendon healing by inhibiting inflammation in vivo and in vitro [10, 21]. Moreover, a study also reported that components in Exos vesicles play a regulatory role in tendon healing [26].

The miRNA has been found to affect mRNA stability and transcription by complementing with mRNA to inhibit its translation or degrade it, thus regulating the expression of target genes [27]. Some studies have shown Exos are enriched in miRNAs, and miRNAs carried by Exos can mediate the communication between tenocytes, e.g., a microarray conducted by Cai et al. demonstrated that miR-499 was aberrantly up-regulated in tendinopathy samples [28]. Similarly, Cui et al. suggested that miR-21-5p inside the macrophage-derived Exos helped tendon healing by targeting Smad7 [26]. The findings of this study are novel, where miR-337-3p over-expressing TDSC-Exos were internalized by tenocytes, which translated into significant tenocyte function recovery via promoting proliferation, migration, and invasion, which was in line with previous studies. Furthermore, CASP3, an apoptosis pathway-related gene, was verified to be a miR-337-3p downstream gene [29]. Effects of Exos (miR-337-3p) on proliferation, migration, invasion, as well as apoptosis of tenocytes were evidently reversed by overexpressing CASP3, suggesting that Exos (miR-337-3p) suppressed tenocyte apoptosis by inhibiting CASP3, providing an alternative therapeutic option in tendon healing.

### Strengths and and limitations

Exos containing miRNAs have demonstrated potential for treating different disorders; for instance, M2 microglia-derived Exos exerted a significant neuroprotective effect when injected into the brain of mice with transient cerebral ischemia, while the neuroprotective effects were partially reversed by injection of down-regulated miR-124 M2 microglia-derived Exos [30]. Previous studies have shown that TDSC-Exos promoted early healing of injured tendons of rats using a scaffold of photopolymerizable hyaluronic acid loaded with TDSC-Exos [12]. TSC-Exos were also mixed in gelatin methacryloyl, and the mixture was placed in the tendon defect to promote healing of injured tendon [21]. As one of the limitations of this study, the maintenance of tendon function by Exos (miR-377-3p) has not been explored in vivo. However, based on previous studies, we speculate that Exos (miR-377-3p) may play a role through various ways, such as the support of scaffold materials and gel materials. Nevertheless, it remains unclear whether Exos (miR-377-3p) could function as an effective regulator for tendon healing in vivo, which warrants further studies to understand the specific mechanisms. Furthermore, it is also possible that miR-377-3p exerts reverse regulatory effects by regulating other target genes that protect tenocytes, which still needs to be further studied.

## Conclusion

The study showed that TDSC-derived Exos (miR-377-3p) can protect against apoptosis of tenocytes by suppressing CASP3. Research on TDSC-Exos in tendon healing is currently at an early stage, and our data could offer insights into the future use of Exos in treating tendon injuries.

### Electronic supplementary material

Below is the link to the electronic supplementary material.

Supplementary Material 1

Supplementary Material 2

## Data Availability

The datasets used and/or analysed during the current study are available from the corresponding author on reasonable request.
