# Supplementary material for: Exosomes derived from mir-337-3p over-expressing tendon stem cells protect against apoptosis of tenocytes via targeting caspase3
Source: BMC Musculoskelet Disord. 2024 Jul 19;25:561. doi: 10.1186/s12891-024-07691-9 (PMC11264700; doi:10.1186/s12891-024-07691-9)
Supplement: Supplementary file 2 — Supplementary Material 2 [file 12891_2024_7691_MOESM2_ESM.docx]

| Figure 2C | Cell extract | Exo(NC) | Exo(miR-337-3p) |
| --- | --- | --- | --- |
| CD9 22kd | 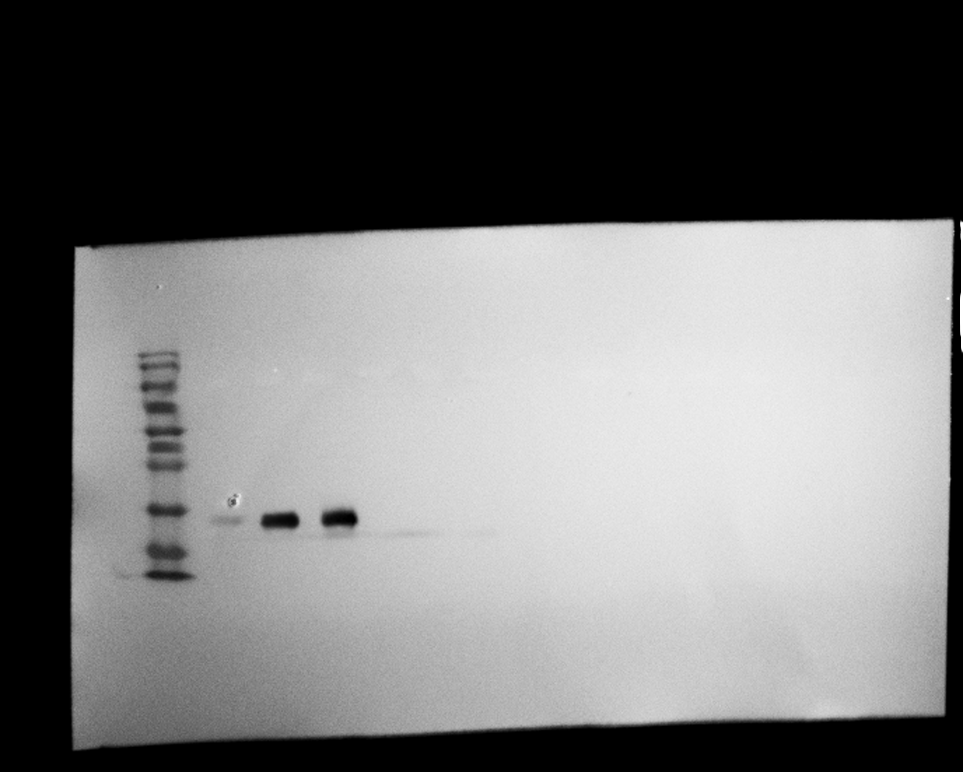 | | |
| CD81 25kd | 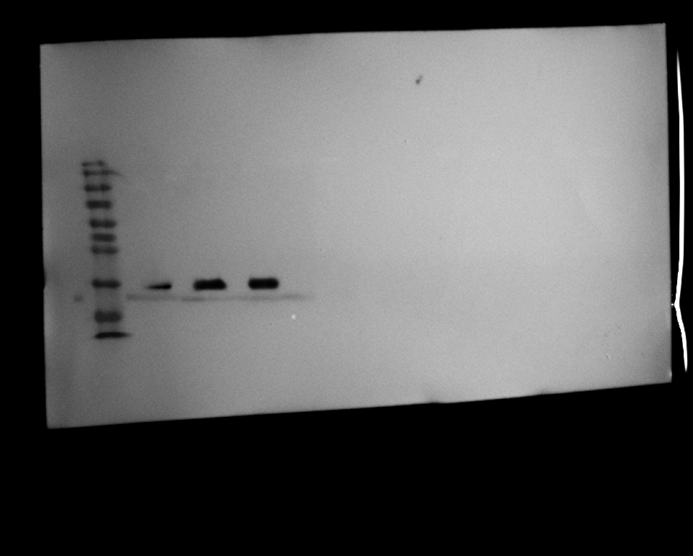 | | |
| HSP70 70kd | 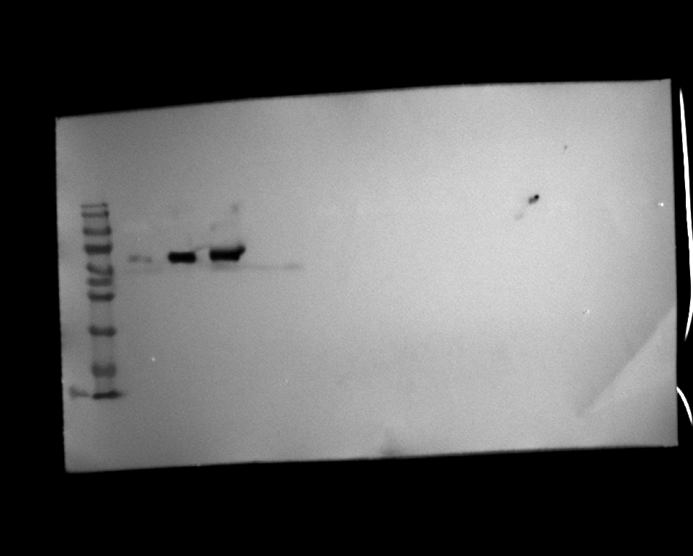 | | |

| Figure 3G | Exo(NC) | Exo(miR-337-3p) |
| --- | --- | --- |
| Bax 21kd | 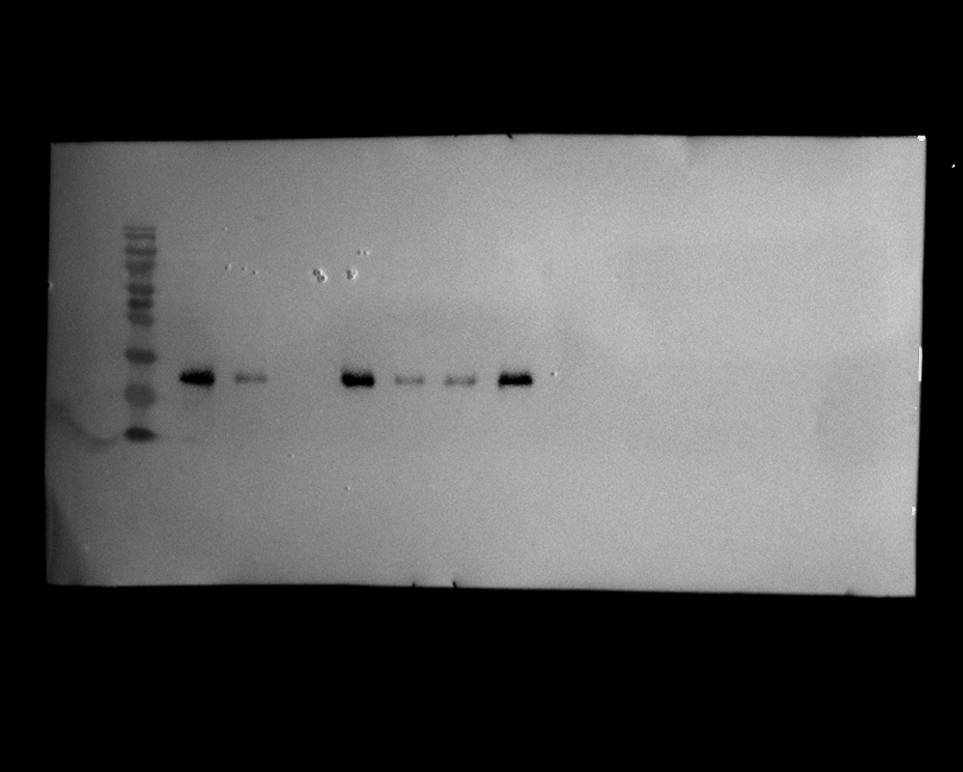 | |
| Bcl2 26kd | 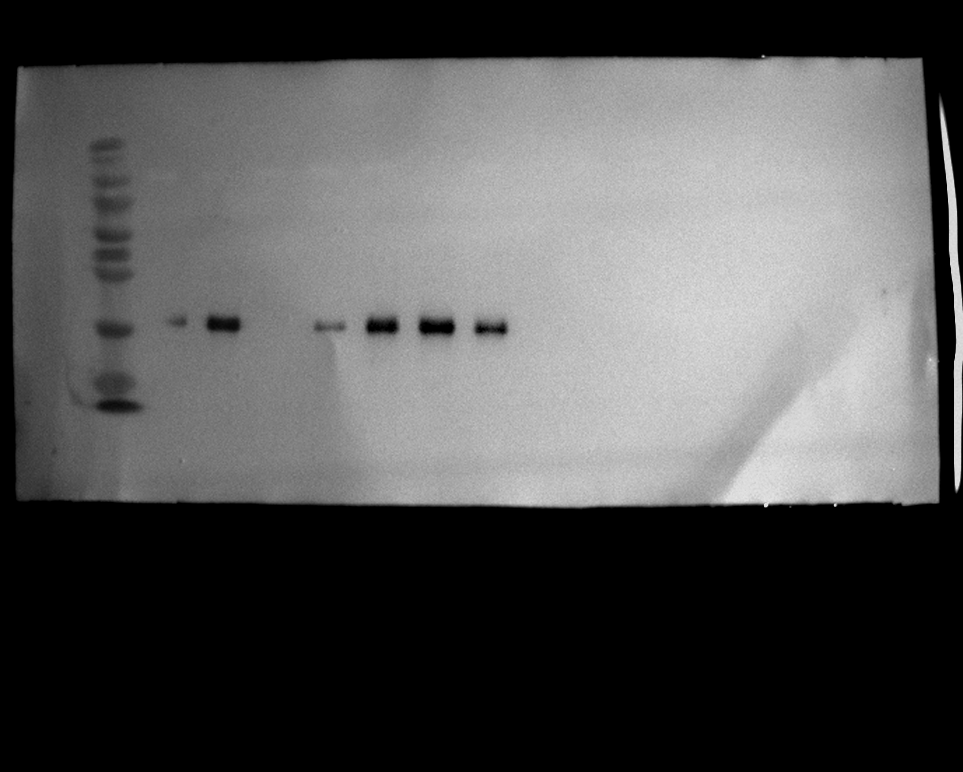 | |
| CL-CASP3 32kd | 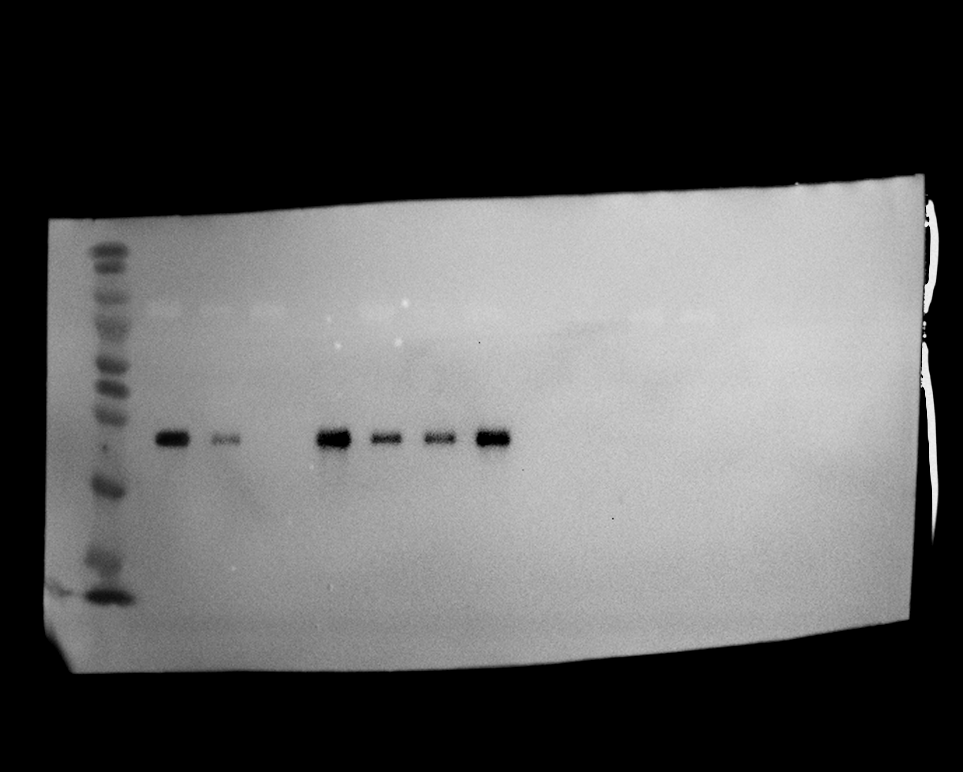 | |
| GAPDH 36kd | 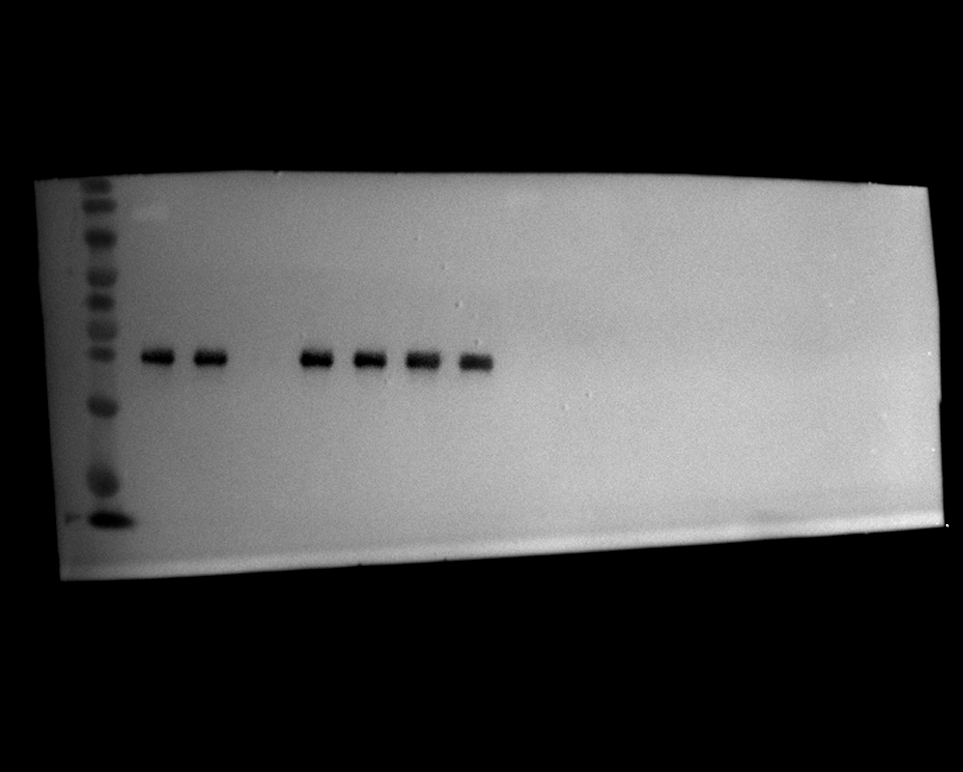 | |

| Figure 4F | Exo(NC) | Exo(miR-337-3p) | Exo(miR-337-3p)+oe-nc | Exo(miR-337-3p)+oe-CASP3 |
| --- | --- | --- | --- | --- |
| Bax 21kd | 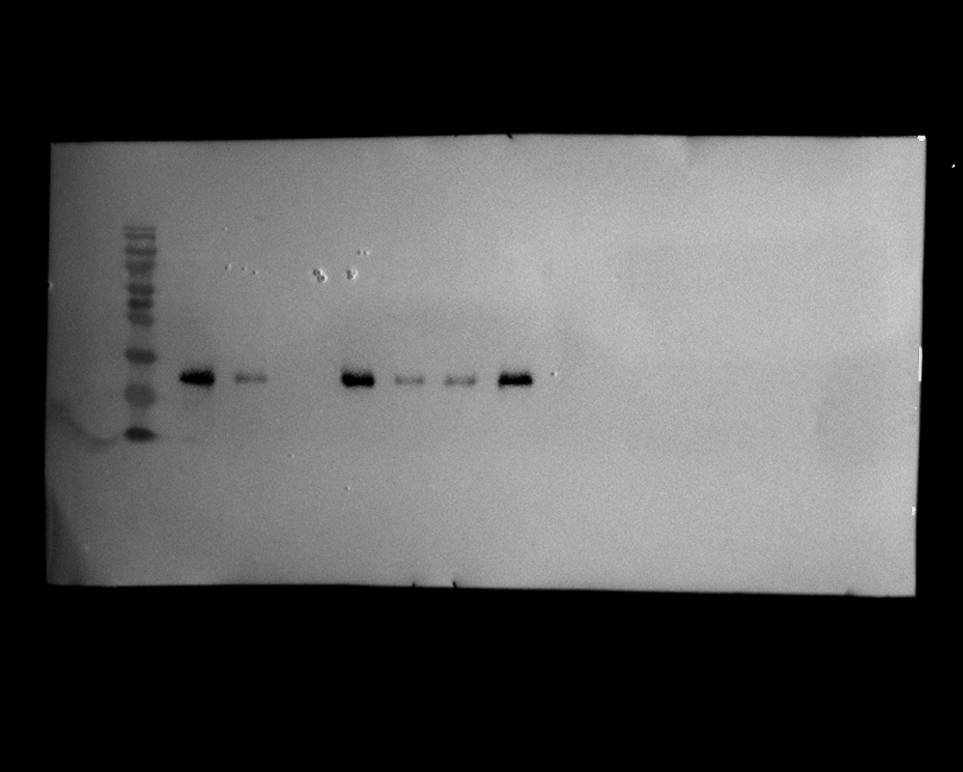 | | | |
| Bcl2 26kd | 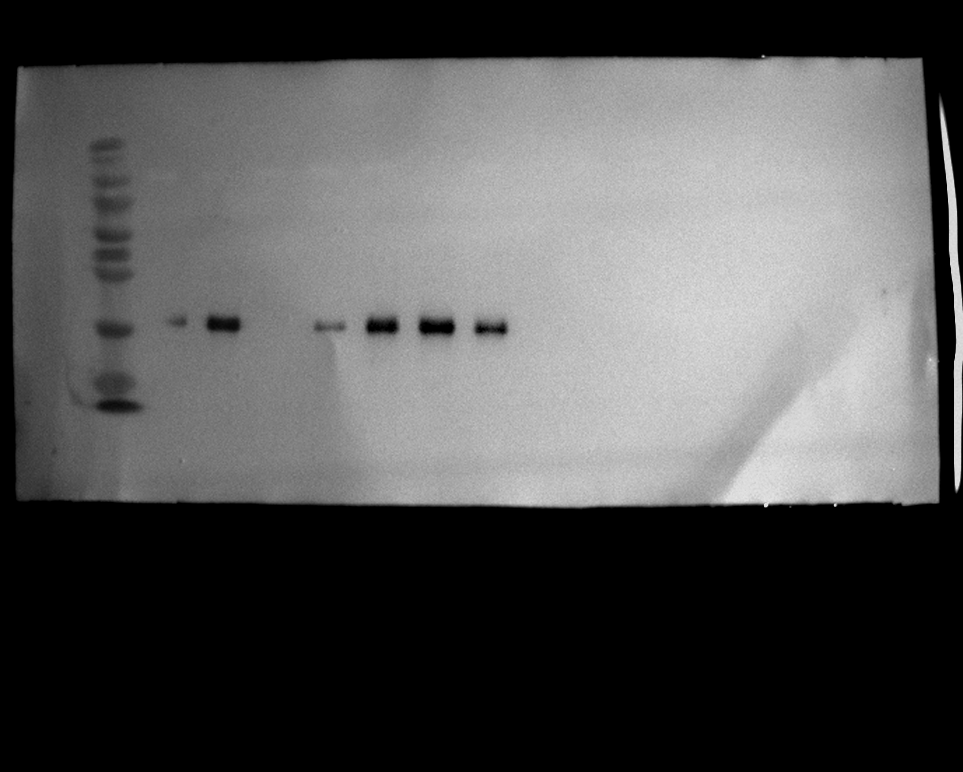 | | | |
| CL-CASP3 32kd | 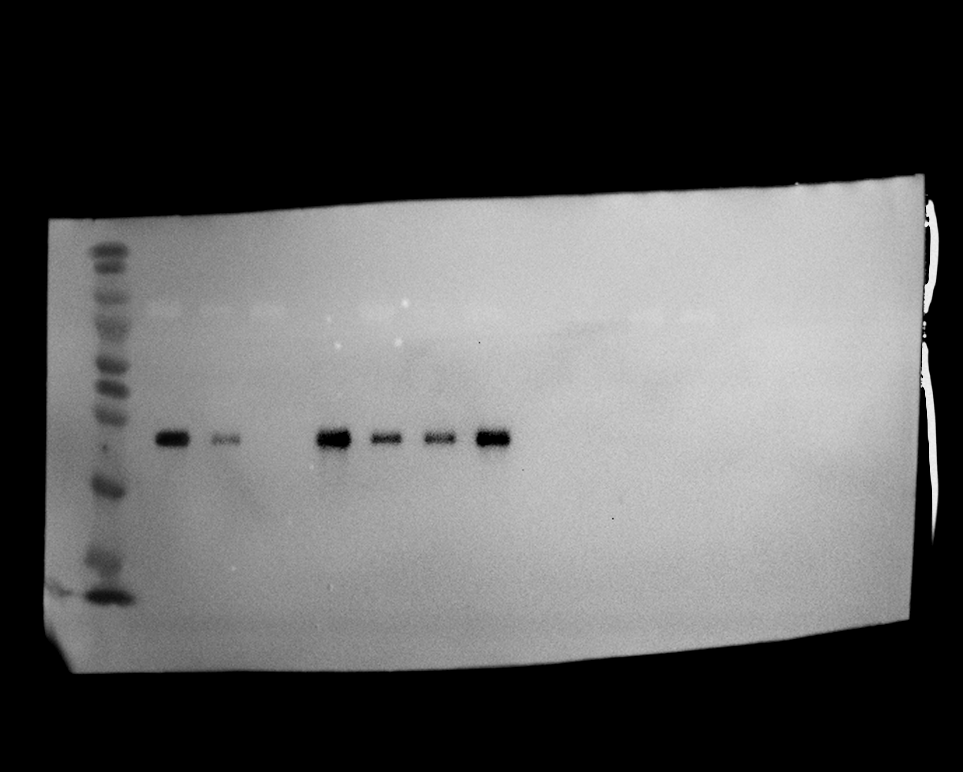 | | | |
| GAPDH 36kd | 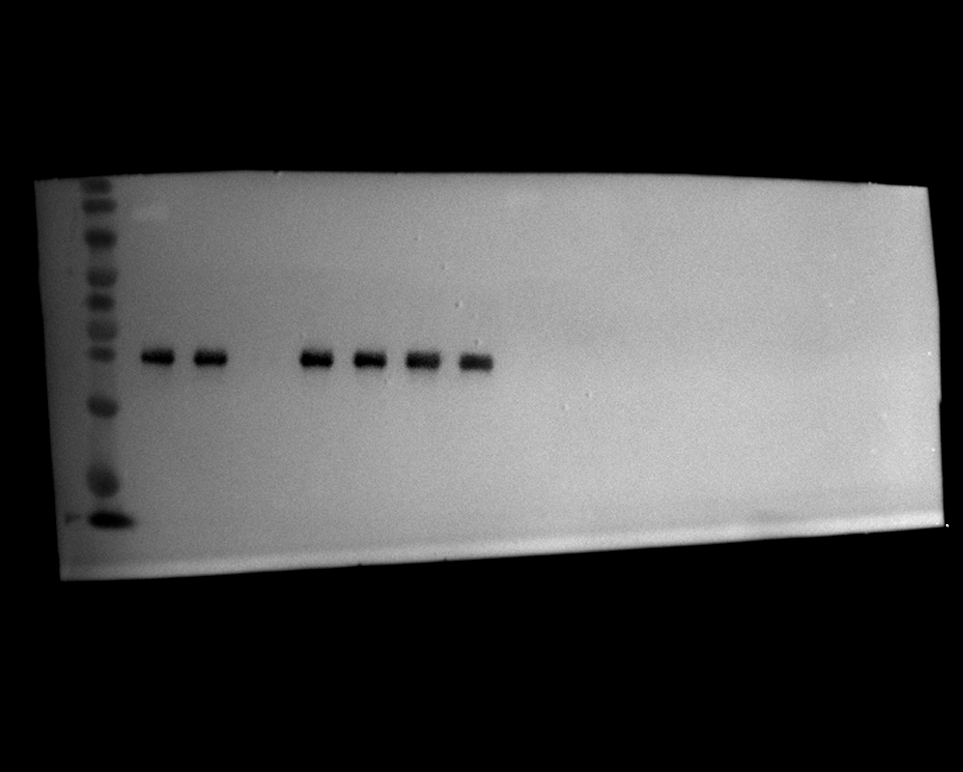 | | | |
